# Phenotypic and Genotypic Comparison of Epidemic and Non-Epidemic Strains of *Pseudomonas aeruginosa* from Individuals with Cystic Fibrosis

**DOI:** 10.1371/journal.pone.0143466

**Published:** 2015-11-23

**Authors:** Jessica Duong, Sean C. Booth, Nathan K. McCartney, Harvey R. Rabin, Michael D. Parkins, Douglas G. Storey

**Affiliations:** 1 Department of Biological Sciences, University of Calgary, Calgary, Alberta, Canada; 2 Department of Microbiology, Immunology and Infectious Diseases, University of Calgary, Calgary, Alberta, Canada; 3 Department of Medicine, University of Calgary, Calgary, Alberta, Canada; UC Berkeley, UNITED STATES

## Abstract

Epidemic strains of *Pseudomonas aeruginosa* have been found worldwide among the cystic fibrosis (CF) patient population. Using pulse-field gel electrophoresis, the Prairie Epidemic Strain (PES) has recently been found in one-third of patients attending the Calgary Adult CF Clinic in Canada. Using multi-locus sequence typing, PES isolates from unrelated patients were found to consistently have ST192. Though most patients acquired PES prior to enrolling in the clinic, some patients were observed to experience strain replacement upon transitioning to the clinic whereby local non-epidemic *P*. *aeruginosa* isolates were displaced by PES. Here we genotypically and phenotypically compared PES to other *P*. *aeruginosa* epidemic strains (OES) found around the world as well as local non-epidemic CF *P*. *aeruginosa* isolates in order to characterize PES. Since some epidemic strains are associated with worse clinical outcomes, we assessed the pathogenic potential of PES to determine if these isolates are virulent, shared properties with OES, and if its phenotypic properties may offer a competitive advantage in displacing local non-epidemic isolates during strain replacement. As such, we conducted a comparative analysis using fourteen phenotypic traits, including virulence factor production, biofilm formation, planktonic growth, mucoidy, and antibiotic susceptibility to characterize PES, OES, and local non-epidemic isolates. We observed that PES and OES could be differentiated from local non-epidemic isolates based on biofilm growth with PES isolates being more mucoid. Pairwise comparisons indicated that PES produced significantly higher levels of proteases and formed better biofilms than OES but were more susceptible to antibiotic treatment. Amongst five patients experiencing strain replacement, we found that super-infecting PES produced lower levels of proteases and elastases but were more resistant to antibiotics compared to the displaced non-epidemic isolates. This comparative analysis is the first to be completed on a large scale between groups of epidemic and non-epidemic CF *P*. *aeruginosa* isolates.

## Introduction

*Pseudomonas aeruginosa* is the principal pathogen in adult cystic fibrosis (CF) patients [1]. Infection with *P*. *aeruginosa* has been associated with both acute and chronic infections, whereby chronic respiratory infections punctuated by acute pulmonary exacerbations ultimately lead to accelerated clinical decline [2]. As airways infection is the leading cause of morbidity and mortality in the CF population [3], understanding the pathogenesis of *P*. *aeruginosa* in CF is of paramount importance.

Because of its wide distribution in nature and particularly in hospitals, it was presumed that CF patients independently acquired *P*. *aeruginosa* strains from their environment [4, 5]. Therefore, each patient was believed to harbor lineages of their own unique (genetically distinct or non-clonal) strains. While most CF patients are colonized with unique strains, clonal epidemic strains have emerged and become widespread amongst unrelated patients [6]. DNA fingerprinting technology has identified epidemic strains in Denmark (DK1, DK2) [7, 8], the United Kingdom (LES, MES, Md1) [9, 10, 11], Australia (AUST-01, AUST-02, AUST-03) [12, 13, 14], the United States (Houston-1) [15], and Canada (Strain A, Strain B, PES) [16, 17].

Our group recently described the Prairie Epidemic Strain (PES), a novel transmissible strain in the Calgary Adult CF Clinic (CACFC) [17]. Using pulse-field gel electrophoresis (PFGE), this strain has been detected since 1980, with a prevalence of 22–37% over three decades of patients transitioning to the adult program. PES infection has been associated with increased rates of lung function decline and progression to end-stage lung disease [18]. PES has not been found in extensive environmental surveys or in other non-CF associated infections confirming its designation as an epidemic CF strain [19]. Parkins *et al*. [17] demonstrated that PES had higher levels of antibiotic resistance against ceftazidime, ciprofloxacin, and tobramycin compared to local non-epidemic isolates. Moreover, PFGE and multi-locus sequence typing (MLST) found that five patients in our cohort had experienced strain replacement by PES. In each of these situations, patients chronically infected with a local non-epidemic isolate of *P*. *aeruginosa* were super-infected with PES, with only PES detected despite subsequent detailed sampling.

In this study, a comparative analysis of PES, other *P*. *aeruginosa* epidemic strains (OES) and local non-epidemic *P*. *aeruginosa* isolates was conducted in order to assess their relative pathogenic potential. We hypothesized that epidemic strains including PES and OES may share characteristics that would enable phenotypic distinction from local non-epidemic isolates. Here classical virulence factors, biofilm formation, growth, and antibiotic susceptibility were measured to determine if these factors differ between the groups. The virulence factors tested included proteases–used to degrade fibrin and collagen to invade host tissues [20], elastases–specifically break down elastin [20], lipases–hydrolyze triglycerides to access fatty acids [21], hemolysins–lyse erythrocytes [20], swarming–a means of coordinated motility mediated by the flagellum and pili [22], and swimming–motility mediated only by the flagellum [22]. We also measured antibiotic susceptibility against 4 classes of antibacterial drugs: aminoglycosides (tobramycin), fluoroquinolones (ciprofloxacin), cephalosporins (ceftazidime), and carbapenems (meropenem). These assays were chosen based on their unambiguous measurements and high-throughput capacity.

We also performed a longitudinal assessment of phenotypic traits of isolates collected from a subset of patients who transitioned to the CACFC (aged 18) and isolates collected more recently (at the time of the study) to understand the natural evolution of infecting isolates over time in patients experiencing strain replacement.

## Materials and Methods

### Strain Collection

One hundred and eighteen CF clinical *P*. *aeruginosa* isolates and the reference strain PAO1 were used in the study. The sample collection was composed of 32 PES (derived from 10 patients), 35 OES (including 6 LES from the United Kingdom, 2 Md1, 1 MES, 3 AUST-01, 2 AUST-02, 1 AUST-03, 1 AUST-04, 1 P42 (AUST-06), 10 Strain A (LES from Canada), and 8 Strain B), and 51 local non-epidemic isolates (derived from 18 patients). The PES and local non-epidemic isolates were obtained from the prospectively maintained and inventoried CACFC Biobank–a comprehensive repository established since 1978 that includes every bacterial pathogen isolated from an individual with CF. For the longitudinal assessment of local isolates, samples were taken at early (at enrollment at age 18) and late (most recent at the time of the study, or prior to transplant/death/loss of follow up) time points. Collection and analysis of *P*. *aeruginosa* from the CACFC Biobank was granted by the Conjoint Health Regional Ethics Board (REB15-0854).

### Multi-locus sequence typing (MLST)

The protocol was adapted from Curran *et al*. [23] and Beaudoin *et al*. [24]. Briefly, seven housekeeping genes (*acsA*, *aroE*, *guaA*, *mutL*, *nuoD*, *ppsA*, *trpE*) were PCR amplified using the amplification primers listed in the S1 Table. PCR conditions were adapted from Korbie and Mattick [25]. PCR products and sequencing primers were sent to Macrogen Inc. to sequence both the forward and reverse strands with the data being uploaded to pubmlst.org [26] for allele type (AT) and sequence type (ST) assignments. Suspected novel ATs were sequenced twice to validate findings.

### Virulence Assays

Bacteria were grown in tryptic soy broth (TSB; Difco, Sparks, MD) for 16–18 hours in a 37°C shaking incubator. Unless otherwise stated, all assays were performed as 3 independent trials, with 3 replicates per trial, resulting in 9 measurements per trait, per isolate. For the protease, elastase, and lipase assays, overnight cultures were standardized to an OD_600_ of 0.3 and 3μl were spotted onto the plates. The plates were incubated at 37°C for 24 hrs (protease), 48 hrs (lipase), or 24 hrs with an additional 48 hrs at 4°C (elastase). The resulting diameter of the zone of clearance was measured. For the swarm and swim assays, a sterile toothpick was used to pick up a single colony from a streak plate and inoculated onto the centre of the agar plate. The plates were incubated at 37°C for 48 hrs (swarm) or 25°C for 72 hrs with additional humidity (swim) and the radius from the point of inoculation was measured.

#### Protease

Procedure was adapted from Sokol *et al*. [27]. Briefly, brain heart infusion (BHI; BBL, Sparks, MD) was dissolved in distilled water (37% wt/vol), poured into a 15 kDa membrane (Spectra, Rancho Dominguez, CA), and dialyzed against 500mL of distilled water at 4°C overnight. This dialysate was combined with 1.5% (wt/vol) skim milk (Difco, Sparks, MD), and 1.5% (wt/vol) noble agar (Sigma-Aldrich, Oakville, ON) to make the agar plates.

#### Elastase

Protocol was adapted from Rust *et al*. [28]. Reverse elastin agar plates were comprised of two layers. The base was comprised of 0.8% (wt/vol) nutrient broth (Difco, Sparks, MD) and 2% (wt/vol) noble agar (Sigma-Aldrich, Oakville, ON) dissolved in distilled water with the pH adjusted to 7.5. The top layer contained 0.8% (wt/vol) nutrient broth (Difco, Sparks, MD), 2% (wt/vol) noble agar (Sigma-Aldrich, Oakville, ON), and 0.5% (wt/vol) elastin from bovine neck ligament (Sigma-Aldrich, Oakville, ON) dissolved in distilled water with the pH adjusted to 7.5.

#### Lipase

Agar plates were prepared according to Lonon *et al*. [21]. The plates were comprised of 1% (wt/vol) peptone (Bacto, Sparks, MD), 0.5% (wt/vol) NaCl (EMD Inc., Mississauga, ON), 0.01% (wt/vol) CaCl_2_•2H_2_O (Sigma-Aldrich, Oakville, ON), 1.5% (wt/vol) noble agar (Sigma-Aldrich, Oakville, ON), and 1% (vol/vol) Tween 80 (Sigma-Aldrich, Oakville, ON) dissolved in distilled water.

#### Swarm

Protocol was adapted from Kohler *et al*. [22]. The agar plates were made using 0.2% (wt/vol) glucose (Sigma-Aldrich, Oakville, ON), 0.5% (wt/vol) noble agar (Sigma-Aldrich, Oakville, ON), 0.05% (wt/vol) glutamic acid (Sigma-Aldrich, Oakville, ON), 0.024% (wt/vol) MgSO_4_ (Sigma-Aldrich, Oakville, ON), and 10% (vol/vol) 10X M8 salt [6% (wt/vol) Na_2_HPO_4_ (Sigma-Aldrich, Oakville, ON), 3% (wt/vol) KH_2_PO_4_ (Sigma-Aldrich, Oakville, ON), 0.5% (wt/vol) NaCl (EMD Inc. Mississauga, ON) in distilled water], in distilled water.

#### Swim

Plates consisted of 0.3% Luria-Bertani agar and the protocol was adapted from Murray and Kazmierczak [29].

#### Hemolysis

*P*. *aeruginosa* was qualitatively assessed for the ability to lyse erythrocytes collected from sheep blood. Bacterial colonies were passaged from a streak plate onto 5% sheep blood TSB agar plates (Dalynn Biologicals, Calgary, AB). The plates were incubated at 37°C for 24 hours and the pattern of hemolysis was measured as beta (complete lysis), alpha (incomplete lysis), or gamma (no lysis). In this instance, only 3 replicates of each isolate were completed.

### Biofilm Assay

#### Biofilm Biomass

This assay adapted the protocol found in Ceri *et al*. [30]. Cultures were normalized to an OD_600_ of 0.001 in TSB and grown in a minimal biofilm eradication concentration (MBEC) plate (Innovotech Inc., Edmonton, AB) that consisted of a lid with 96 pegs and an accompanying 96-well Nunclon Delta Surface plate (Thermo Scientific, Roskilde, Denmark). The biofilm was grown for 24 hours in a 37°C shaking incubator with extra humidity. The lid containing the pegs was subsequently removed and stained with 1% crystal violet (Sigma-Aldrich, Oakville, ON), washed thrice with phosphate buffered saline (0.1% (wt/vol) Na_2_HPO_4_ (Sigma-Aldrich, Oakville, ON), 0.02% (wt/vol) KCl (Sigma-Aldrich, Oakville, ON), 0.8% (wt/vol) NaCl (EMC Inc. Mississauga, ON), 0.02% (wt/vol) KH_2_PO_4_ (Sigma-Aldrich, Oakville, ON) in distilled water) and stripped into methanol (EMD Inc., Mississauga, ON) in order to measure biomass at an OD_550_ in a Perkin Elmer Victor X4 plate reader.

#### Biofilm Growth

A second MBEC device was set up as stated above. In this case, the pegs were removed and placed in a 0.85% saline solution. The biofilms that formed on the pegs were disrupted using sonication for 5 mins (5510 Branson). Serial dilutions were performed to determine viable cell counts on TSB agar plates.

### Planktonic Growth

The bottom half of the MBEC device was measured for planktonic growth at an OD_600_ in a Perkin Elmer Victor X4 plate reader.

### Mucoidy

*P*. *aeruginosa* strains were qualitatively assessed for mucoidy. Bacterial colonies were streaked onto *Pseudomonas* isolation agar (PIA; Difco, Sparks, MD) and incubated at 37°C for 48 hrs. A slimy phenotype was rated as positive for the presence of mucoid-producing cells. In this instance, only 3 replicates of each isolate were assayed.

### Antibiotic Susceptibility

The Kirby-Bauer disc diffusion test [31] was used to measure activity of ceftazidime (Oxoid, Nepean, ON), ciprofloxacin (Oxoid, Nepean, ON), meropenem (Oxoid, Nepean, ON), and tobramycin (Oxoid, Nepean, ON). Three independent trials of each antibiotic were conducted, with the mean value reported. Resistance breakpoint values were interpreted as found in the European Committee on Antimicrobial Susceptibility Testing (EUCAST) clinical breakpoint table [32]. Resistant breakpoint values were: tobramycin < 16 mm, ceftazidime < 16 mm, ciprofloxacin < 22 mm, meropenem < 18 mm.

### Hierarchical Clustering Analysis

Cluster 3.0 was used to group the phenotypic traits based on the unweighted pair group method with arithmetic mean. The gene cluster, correlation uncentered, and centroid features under the hierarchical tab were used. For each phenotypic assay, the data was mean-centered and scaled to unit-variance in order to account for the differences in dynamic range and variance of each trait to allow the variables to be considered together. Java Treeview was used to build the dendrogram.

### Statistical Analysis

Kolmogorov-Smirnov (KS) and Mann-Whitney U (MWU) tests as well as Benjamini-Hochberg false-discovery corrections were performed in ‘R’ (www.R-project.org). For all tests a p-value <0.05 was considered significant and the multiple testing problem was accounted for by using the Benjamini-Hochberg step-up procedure [33] to control the false discovery rate (FDR). Briefly, FDR was calculated by ranking p-values and multiplying each p-value by the number of tests performed and dividing them by the rank. The largest p-value still under 0.05 becomes the new p-value cutoff for significance, which are reported for each group of tests. MWU tests with FDR control were used for the longitudinal study to compare isolates from different time points from the same patient for all phenotypic traits, but a paired test was not used as the number of strains isolated at each time (early or late) did not always match.

KS tests were used to determine whether values from one group were larger or smaller than the other group. The KS test is based on the empirical cumulative distribution function (ECDF), which indicates the cumulative percentage of isolates from each group type with at least that much activity, as determined by the x-axis for each phenotypic trait. The KS test was used as it does not assume normality of data, nor homoscedasticity, and is insensitive to log transformation. Separate KS tests were performed that checked whether the ECDF of each group listed in the table was respectively larger or smaller than that of the second group.

Chi-square tests were employed to determine whether there were significant differences between the groups in the mucoidy and hemolysis assays.

## Results

### MLST of Epidemic and Non-Epidemic *P*. *aeruginosa*

Using MLST, we were able to validate our findings from the PFGE analysis (refer to Parkins *et al*. [17]) and confirm that PES are indeed clonal. Full allele types (ATs) and sequence types (STs) are provided in the S2 Table. In 31/32 (97%) of the PES isolates an ST of 192 was found. In one isolate, 6/7 loci matched that of ST192 but a novel AT was found at *guaA* (AT132) due to single nucleotide difference from AT7. This resulted in a new ST1495 designation but a difference at only 1 locus is not enough to differentiate isolates [34] and it was therefore categorized as PES.

As for the OES, none of the isolates were found to type as ST192 that was displayed by PES. The most related OES to PES were two strain A’s isolates (ST683) and one Strain B isolate (ST New) as each shared 3 allele types with the PES sequence type (S2 Table). LES/Strain A (hereafter referred to as LES) isolates were found to have two sequence types–ST146 and ST683, which have previously been identified [16, 35]. These two sequence types differed at the *ppsA* locus but ST146 was the dominant ST as it was present in 13/16 (81%) of the isolates. Our collection also included 2 Md1 isolates and both were ST148 clones [35]. The MES was found to have a novel ST but it matched 6/7 allele types of a previously identified Manchester strain [36]. The other Canadian epidemic strain, Strain B, had two STs but ST439 [16] was found in 6/8 (75%) of the isolates. The Australian strains had five STs, but the strains in this subset are known to be genetically distinct and coincided with previously published work [37].

In typing the 51 local non-epidemic isolates (also referred to as local isolates) from 18 patients, we found 24 different STs with 12 being novel types unique to the CACFC (S2 Table). We observed that patients who harbored these non-epidemic isolates tended to have the same or related strains at subsequent time points (S2 Table). In particular, 6 patients were stably colonized with local isolates (patients A2, A9, A14, A40, A51, A52) for up to 17 years. Moreover, 6 other patients (A18, A34, A35, A64, A85, A129) harbored multiple isolates that were the same local clone or lineages of the clone since nucleotide sequence changes were found at 1–3 ATs (S2 Table). In three of our patients (A18, A52, A129) we found stable isolates with a MLST sequence type of ST179 (S2 Table). Moreover, two of our patients (A8, A11) were colonized with ST274 (S2 Table). These sequence types were the only ones other than ST192 to be found in more than one patient.

Six additional patients were observed to undergo strain replacement (S2 Table). Five of these patients (A11, A43, A78, A131, A134) were each initially colonized with local isolates that were subsequently replaced by PES and this replacement occurred in as little as 2 years. One patient (A8) experienced strain replacement of a local non-epidemic isolate by a different non-clonal isolate (S2 Table).

### Clustering of Phenotypic Traits

**Fig 1:**
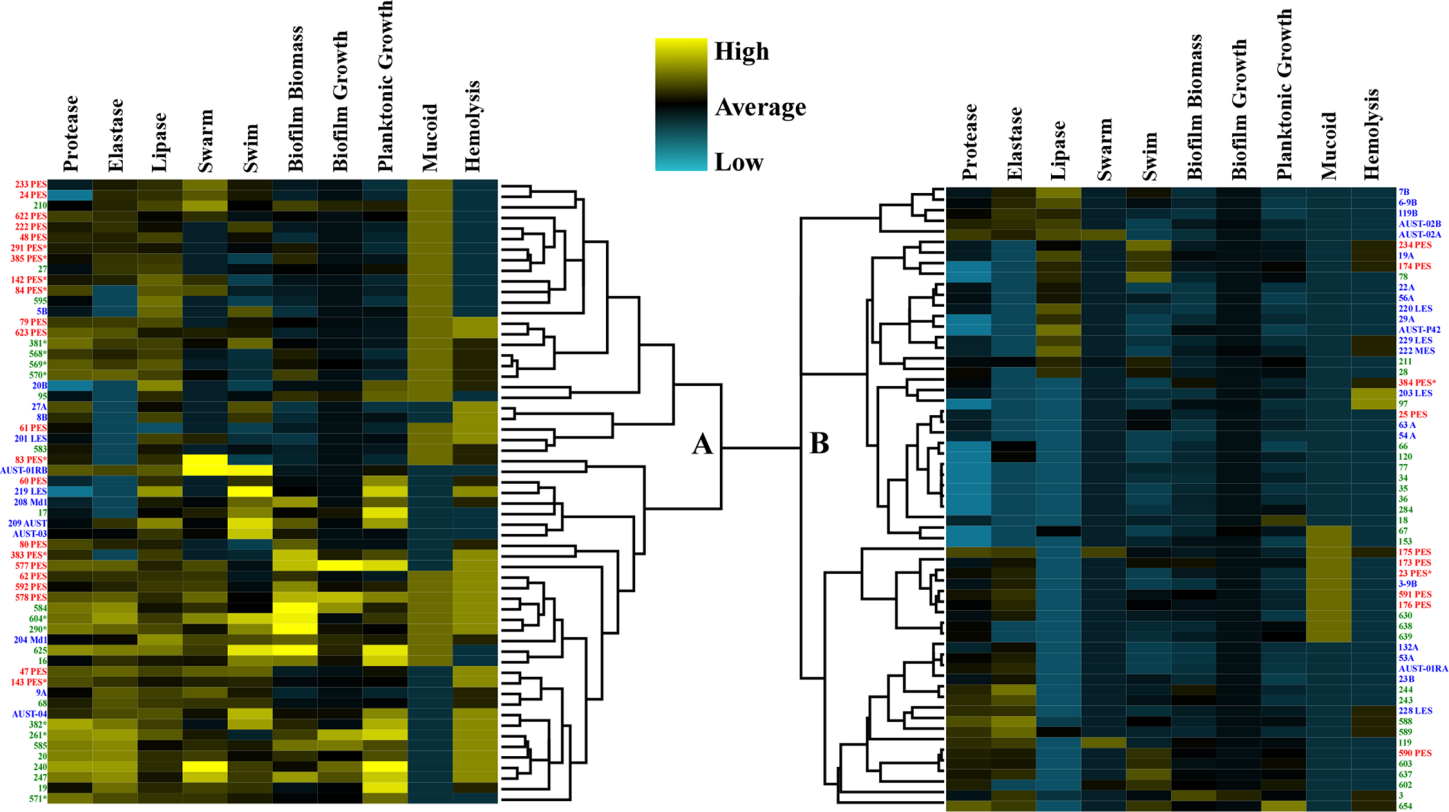
Hierarchical clustering analysis of phenotypic traits of *P*. *aeruginosa* isolates. The dendrogram is split into two main clades (A and B). The mean value for all tests are represented as black boxes, higher than average virulence factor production is indicated in yellow, and lower than average is shown in blue. PES isolates are listed in red text, OES are in blue text, and local isolates are in green text. An asterisk denotes those isolates involved in strain replacement. Values were mean-centered and scaled to unit-variance. Dendrogram was built using Cluster 3.0 that contained a hierarchical feature with the gene cluster, correlation uncentered, and centroid linkage options.

Classical virulence factors were measured for all isolates, including enzymatic activity (protease, elastase, lipase, and hemolysin production), motility (swarm and swim), biofilm formation (biomass and growth as measured by colony forming units (CFUs)), planktonic growth, and mucoid phenotype. *P*. *aeruginosa* isolates were holistically compared based on all phenotypes tested. Hierarchical clustering analysis was used to cluster the isolates based on the similarity of their phenotypic traits. This analysis produced two main clades (Fig 1). Clade A was mainly comprised of isolates expressing higher levels of virulence factors and 22/32 (69%) of PES fell into this group (Fig 1 clade A). Within clade A, PES largely clustered into two groups with four other side by side pairings. Conversely, clade B was comprised of isolates expressing lower levels of virulence factors and only 10/32 (31%) of PES were found in this clade (Fig 1 clade B). Most of the OES clustered in clade B (22/35, 63%) while the local isolates were more evenly distributed since 24/51 (47%) expressed higher activity (clade A) of the phenotypic traits whereas 27/51 (53%) expressed lower activity (clade B) of the factors tested.

We also looked at the clustering of the PES and local isolates that were found in the five patients where strain displacement was observed. Of the displaced local isolates, all were found in clade A with the higher level of virulence determinants (Fig 1). Similarly, 7/9 (78%) of the replacement PES were also found to cluster in clade A.

### Comparison of Phenotypic Traits Between Epidemic and Non-Epidemic Isolates

Based on our cluster analysis, we wanted to determine if there might be phenotypic traits that would distinguish epidemic isolates from non-epidemic isolates. Isolates were categorized into three groups (PES, OES, local isolates) with the OES comprised of various other epidemic strains grouped into a single entity due to their small sample size and to assess if there is a common phenotypic marker among the epidemic strains.

**Fig 2:**
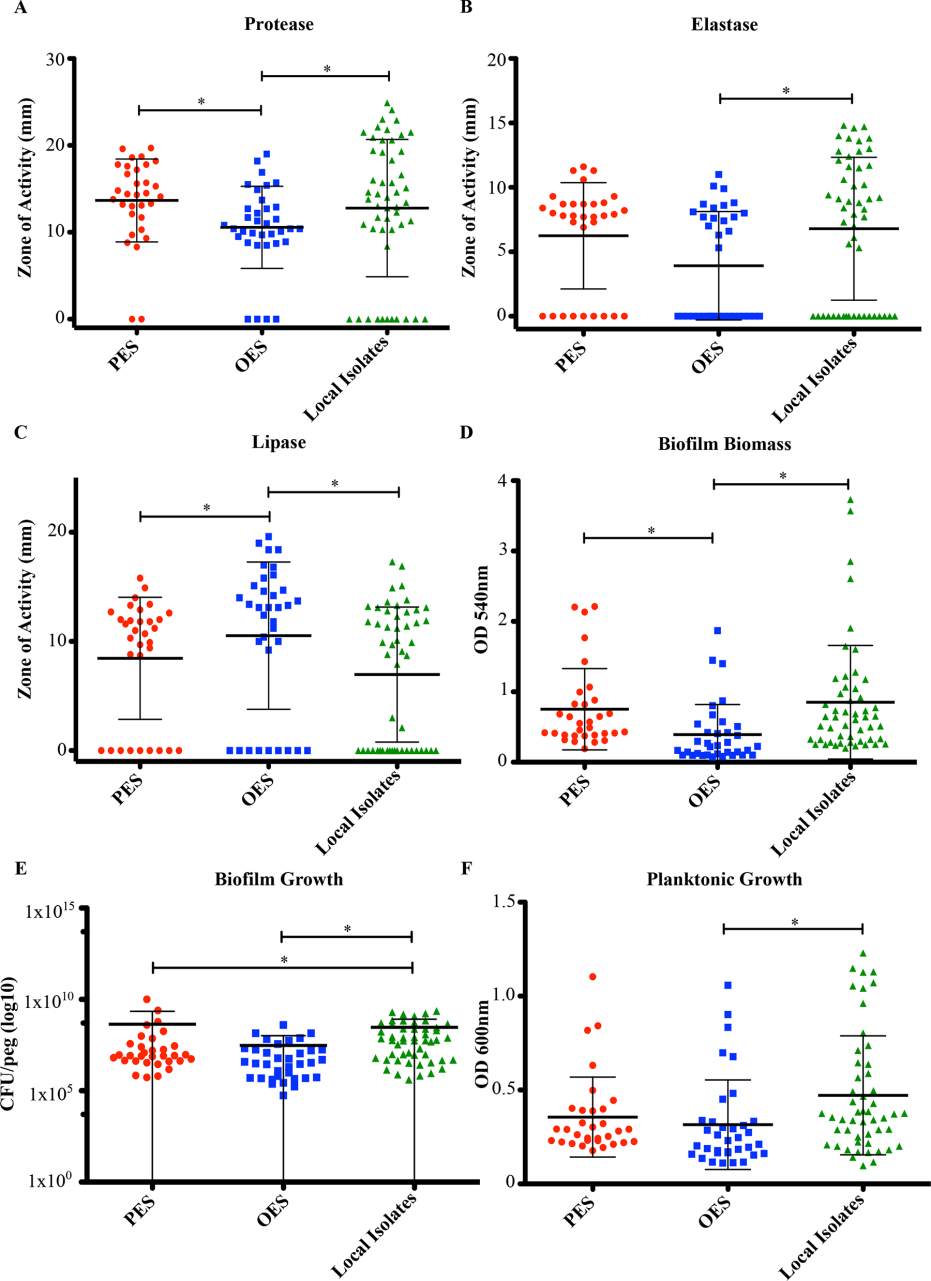
Phenotypic traits of *P*. *aeruginosa* isolates. A) Protease. B) Elastase. C) Lipase. D) Biofilm Biomass. E) Biofilm Growth (log scale). F) Planktonic Growth. Isolates were separated into three groups for comparative analysis: Prairie Epidemic Strain (PES, red), other epidemic strains (OES, blue), and local non-epidemic isolates (green). Each circle depicts the mean activity of one *P*. *aeruginosa* isolate. The horizontal line indicates the mean activity of each group while the error bars displays the standard deviation. Significant differences according to the KS test are marked with asterisks.

When we compared the epidemic strains (PES and OES) against the local isolates, we noticed that all three groups had a wide distribution of activity for each trait (Fig 2A–2F). In terms of individual traits few showed significant differences between the epidemic strains and the local isolates (Fig 2A–2F, S1 Fig and S3 Table). The two exceptions were biofilm growth (Fig 2E) and mucoidy (S2A–S2C Fig). Significant differences were found between the epidemic strains (both OES and PES) and the local non-epidemic isolates for biofilm growth (Fig 2E and S3 Table). In terms of mucoidy, 66% of the PES isolates exhibited the mucoid phenotype whereas only 14% of the OES and 37% of the local non-epidemic isolates were mucoid (S2A–S2C Fig). These differences in mucoidy were significantly different between all groups (S4 Table).

**Fig 3:**
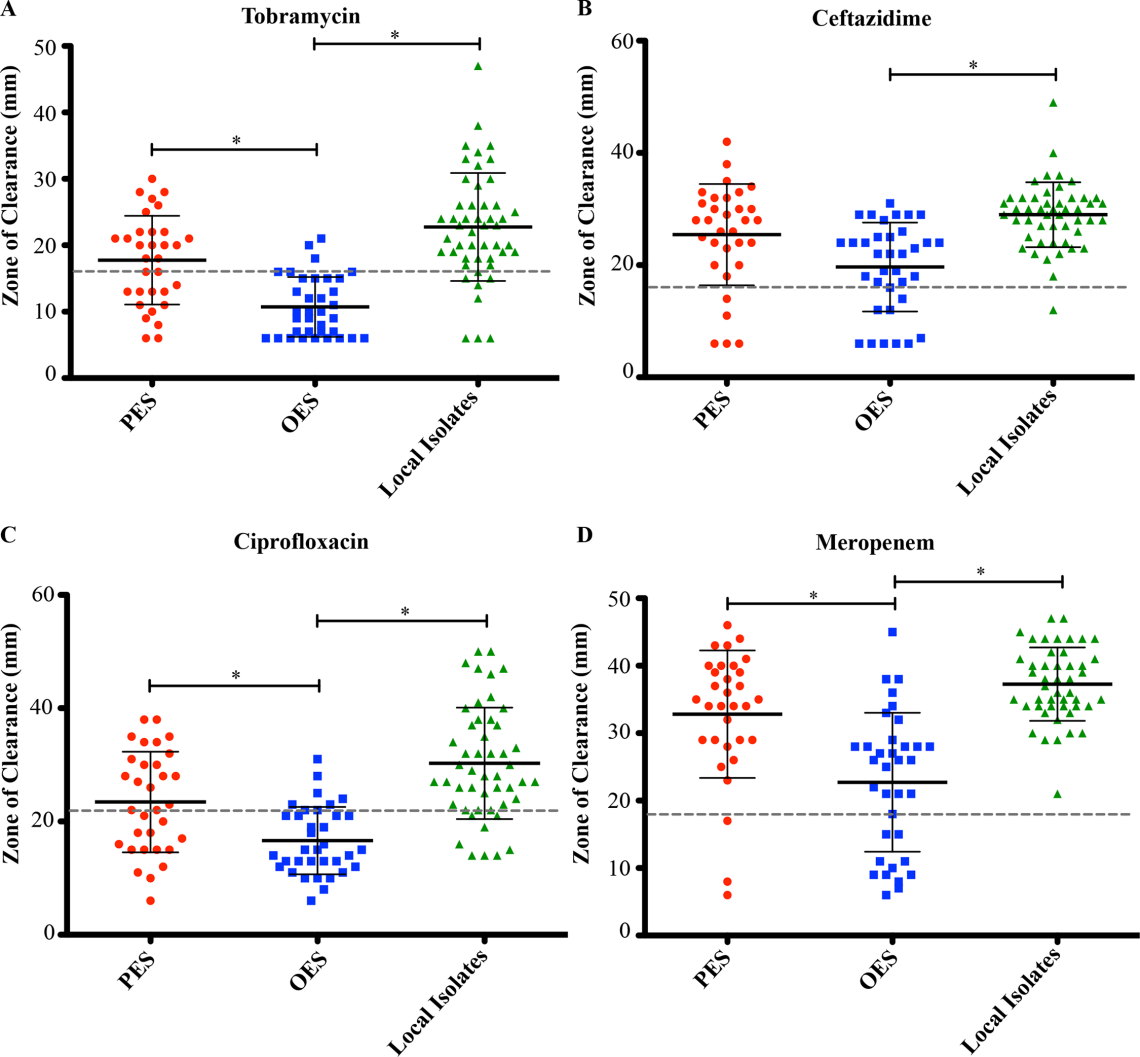
Antibiotic susceptibility testing of *P*. *aeruginosa* isolates. Prairie Epidemic Strain (PES, red), other epidemic strains (OES, blue), and local non-epidemic isolates (green) were assayed for A) tobramycin, B) ceftazidime, C) ciprofloxacin, and D) meropenem susceptibility. Mean susceptibility values of Kirby-Bauer zone sizes were measured for each individual isolate. The black bar indicates the mean (+/- standard deviation) of each group whereas the dashed grey line depicts the resistance breakpoint value according to EUCAST standards [32]. Significant differences between the groups according to the KS test are indicated by the asterisk symbols.

The antibiotic susceptibility tests demonstrated that only OES could be differentiated from the local non-epidemic isolates (Fig 3A–3D). The OES were more resistant to all four antibiotics compared to the local isolates, especially against tobramycin (83% of isolates) and ciprofloxacin (77% of isolates) (Fig 3A–3D and S3 Table). Conversely, the local isolates were highly susceptible since only 1 isolate was resistant to ceftazidime and none were resistant to meropenem (Fig 3B and 3D).

Pairwise group comparisons of the activities of all isolates found that the local isolates had the highest mean activity for elastase, biofilm biomass, and planktonic growth (Fig 2B, 2D and 2F). Conversely, OES had the lowest mean activity for all factors tested except for lipase production, where this group had the highest mean level of activity (Fig 2C). Statistical analysis indicated that PES could be differentiated from OES based on their higher protease activity and greater better biofilm biomass but lower lipase activity (Fig 2A, 2C and 2D and S3 Table). This was also true in terms of antibiotic susceptibility where the OES were more resistant than PES for three of the four antibiotics tested (Fig 3A–3D and S3 Table). The only phenotypic characteristic that could separate the two groups was PES having lower numbers of CFUs in the biofilm mode of growth (Fig 2E and S3 Table). Of interest, none of the groups could be differentiated from each other based on the motility (S3 Fig and S3 Table) or hemolysis (S4 Fig and S4 Table) assays.

### A Comparison of Phenotypic Traits of Stable and Strain Replacement Isolates

A longitudinal (early vs. late) assessment of colonizing *P*. *aeruginosa* isolates from individual patients was performed to determine how infecting strain characteristics change over time. These isolates were separated into three groups based on the patient cohort they were derived from: those patients stably infected with PES for at least 6 years (n = 23, early = 10, late = 13), those patients stably infected with non-epidemic local isolates for at least 5 years (n = 38, early = 19, late = 19), and patients that experienced strain replacement in as little as 2 years (n = 18, early = 9, late = 9).

**Fig 4:**
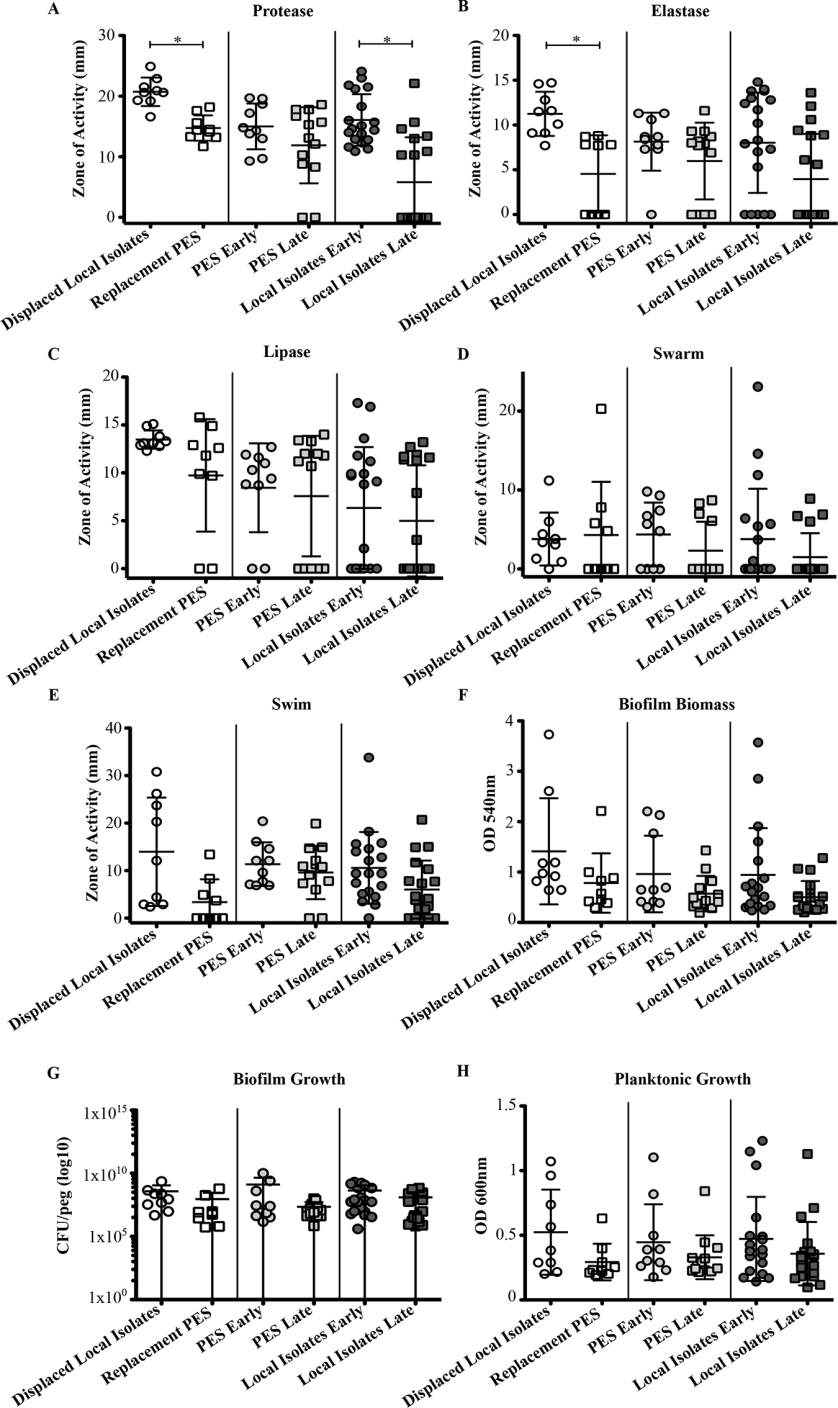
Longitudinal comparison of virulence factors, biofilm formation, and growth of *P*. *aeruginosa* isolates. A) Protease. B) Elastase. C) Lipase. D) Swarm. E) Swim. F) Biofilm Biomass. G) Biofilm Growth (log scale). H) Planktonic Growth. Each data point represents the mean (+/- standard deviation) activity of a single isolate. Circles represent early isolates whereas squares represent late isolates. Three different situations were observed: local isolate displaced by PES via super-infection (white), PES stably colonizing a patient (grey), and local isolate stably colonizing a patient (black). Significant differences between the groups according to the MWU test are indicated by asterisk symbols.

Comparisons of these super-infecting PES isolates demonstrated that relative to the replaced local non-epidemic isolates that they displaced, they tended to be less virulent in general (Fig 4A–4H). Statistical analysis indicated that significant differences between these two subgroups were only found in protease and elastase production assays (S5 Table). In particular, PES produced lower levels of both of these enzymes than the displaced isolates (Fig 4A and 4B). The local isolates showed a similar trend as protease production was significantly different between the early and late groups (Fig 4A and S5 Table). However, for the patients stably colonized with PES, no significant differences were found between the early and late isolates for any of the traits tested (Fig 4A–4H and S5 Table).

**Fig 5:**
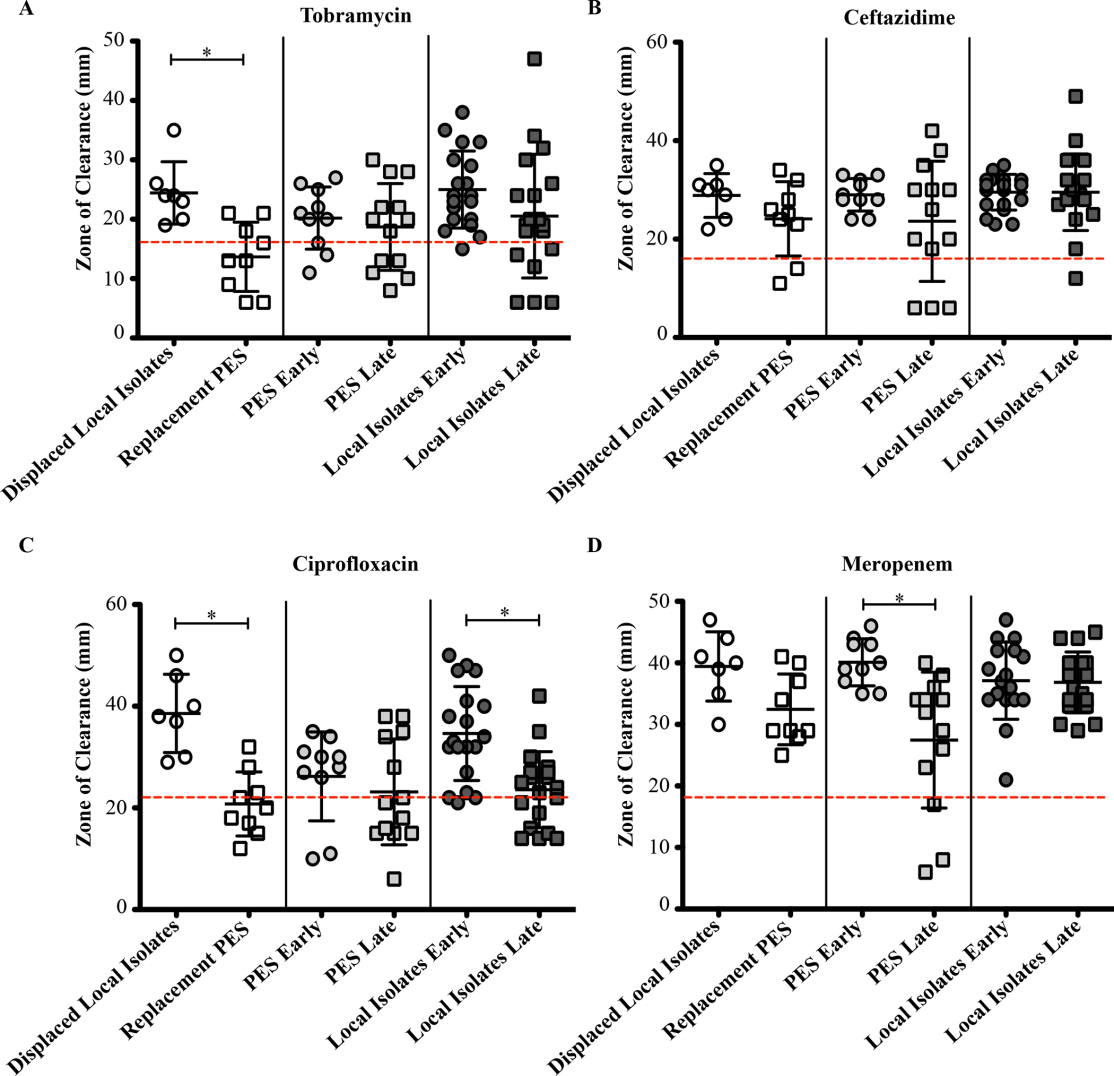
Longitudinal comparison of antibiotic susceptibility of *P*. *aeruginosa* isolates. A) Tobramycin. B) Ceftazidime. C) Ciprofloxacin. D) Meropenem. Each data point represents the mean activity (+/- standard deviation) of a single isolate. Circles represent early isolates whereas squares represent late isolates. Three different situations were observed: local isolate displaced by PES via super-infection (white), PES stably colonizing a patient (grey), and local isolate stably colonizing a patient (black). The dashed red line depicts the EUCAST resistance breakpoints [32]. Significant differences between the groups according to the MWU test are indicated by the asterisk symbols.

A comparison of antibiotic susceptibility between the displaced isolates and PES showed that the displaced isolates were generally more susceptible to antibiotics than the PES that replaced them (Fig 5A–5D). In particular, 67% of PES were resistant to tobramycin and 78% resistant to ciprofloxacin (Fig 5A and 5C). In contrast, none of the displaced isolates were resistant to the antibiotics (Fig 5A–5D). Statistical analysis confirmed that these differences were significant (S5 Table). Likewise the late stable local isolates were significantly more resistant to ciprofloxacin (33%) compared to the early local isolates (5%) (Fig 5C and S5 Table). With the exception of susceptibility to meropenem, the PES stable group did not show any significant difference in activity between the early and late isolates (Fig 5D and S5 Table).

## Discussion

Since the discovery of LES in 1996, the dogma of transmissible and epidemic *P*. *aeruginosa* strains being a rare occurrence has been dispelled [9]. Although most CF patients are colonized with non-epidemic strains of *P*. *aeruginosa*, several epidemic strains have been found around the world amongst the CF population [4, 5]. With the exception of the European LES and Canadian Strain A [16], each epidemic strain is genetically distinct and they are commonly, but not universally, associated with worsened disease progression and/or multi-drug resistance [6]. Currently, it is unknown if this resistance is an intrinsic property or an adaptive response to increased antibiotic pressure [24].

PES is prevalent among CF patients attending the Calgary Adult CF Clinic (CACFC) and amongst patients transferring from other clinics in the Prairie provinces of Canada suggesting broader prevalence [17]. Herein we used MLST to genotype *P*. *aeruginosa* isolates since this technique is unambiguous and reproducible across multiple labs [23]. This work found that the PES (ST192) clones are genetically distinct from the other epidemic strains. Patients that were not infected with PES tended to carry lineages of their own local non-epidemic isolates (S2 Table) that persisted for many years as found in other clinics [18, 38, 39]. Among our patients, MLST identified clones (ST179) in a pair of CF siblings (A18 and A129) (Parkins *et al*. [17] and this study). This clone was also detected in one other unrelated patient (A52). Additionally, ST274 clones were found in two unrelated patients (A8 and A11). These ST179 and ST274 isolates are minor clones common in the local environment that have been detected in a small number of other patients in the clinic and elsewhere [34, 37].

Adaptation to CF airways can be accomplished in a myriad of mechanisms, including loss of virulence factors over time [40–42], biofilm formation [43], and tolerating antibiotic treatment [44]. We initially thought that PES and OES might be phenotypically similar since both have been able to spread throughout CF patient populations. Our results suggest that epidemic strains adapt to CF lungs to cause chronic infections in three key ways–biofilm growth, conversion to the mucoid phenotype, and antibiotic resistance. We found that biofilm growth was the only trait that separated both PES and OES from the local non-epidemic isolates with PES isolates being abundantly mucoid. It is now well established that mucoidy is a result of mutations in the *mucA* gene, which in turn allows for the over-expression of alginate [45]. Though not completely essential for biofilm formation, alginate is commonly found in the extracellular matrix of biofilms [46, 47]. This conversion to the mucoid phenotype serves as an adaptive response to the harsh CF lung environment and patients chronically infected with mucoid isolates tend to have worsened disease progression [48].

The antibiotic susceptibility assays indicated that OES were more resistant than the local isolates. Furthermore, PES isolates that participated in strain replacement were more resistant than their displaced non-epidemic isolate counterparts, especially to tobramycin and ciprofloxacin. However, PES were more likely to be susceptible to antibiotics compared to LES (Parkins *et al*. [17] and this study). The CACFC is conservative in its admistration of antibiotics during routine treatment meaning PES would have less pressure to develop resistance compared to LES and other epidemic *P*. *aeruginosa* strains [49, 50]. Together with the biofilm and mucoidy data, the antibiotic susceptibility analysis suggests that epidemic isolates have a competitive advantage over the local isolates in the CF lungs. This is important in terms of strain replacement. Super-infections refer to secondary infections that have superimposed on a primary infection, whereby the secondary infection dominates and the original strain is no longer detected leading to strain replacement [51]. These super-infection events have been documented with LES with the worry that strain replacement may lead to worsened patient prognosis [51, 52]. Five patients (A11, A43, A78, A131, A134) in this study were initially colonized with a local non-epidemic isolate but were later super-infected with PES ST192 clones. Serial yearly assessments following this event demonstrated total dominance by the super-infecting PES [17]. The longitudinal study was necessary as the hierarchical analysis of all the strains displayed that all of the displaced local isolates and 7/9 of the replacement PES grouped together. This indicated that these isolates had similar virulence profiles and it is likely that strain replacement is a phenomenon that cannot be attributed to differences in virulence traits alone.

We were able to demonstrate that even amongst genetically distinct isolates from the same patient involved in strain replacement that reduction of virulence factors continued to occur. In particular, 4/9 (44%) of the replacement PES did not have elastase activity whereas all the displaced local non-epidemic isolates produced elastase. Elastase production is regulated by the *lasIR* quorum sensing (QS) system [53] implying that PES could have evolved to be QS mutants. Since the bacteria are decreasing their production of virulence factors, they are not eliciting host responses to the same degree and can better evade constant immune surveillance, resulting in the establishment of chronic infections. This phenotype is beneficial as it would aid the bacteria evade host immune responses mediated by polymorphonuclear leukocytes [54] and may even allow the bacteria to better compete. Moreover, the PES stable isolates did not exhibit a significant change of activity between the early and late time points, which was also observed in the hierarchical clustering analysis since PES isolates tended to group together suggesting that they have similar phenotypic profiles. However it is possible that since we are assessing these replacement PES isolates many years after the super-infection event, the original traits enabling super-infection may have been altered or completely lost.

We acknowledge that phenotypic heterogeneity in *P*. *aeruginosa* isolates have been extensively reported in CF literature [55–57]. Even though we randomly selected isolates for this study, we are inferring phenotypes to the entire population. Sampling bias may also exist due to the relatively small number of *P*. *aeruginosa* isolates from the same patient and that other virulence factors, such as pyocyanin [58], siderophore [59], and DNase [60] production, could have been studied that could have served to better differentiate the epidemic and non-epidemic groups. Here we provide a comprehensive assessment of the phenotypic traits and genotypic background of *P*. *aeruginosa* causing chronic infections in CF. This study is the first to compare multiple *P*. *aeruginosa* epidemic and non-epidemic isolates in order to provide insight on strain replacement. The mechanisms that are involved in strain replacement remain largely unknown but our study has demonstrated that virulence factor production is likely not the sole component used. Elucidating these mechanisms will provide a better understanding regarding the rapid spread of *P*. *aeruginosa* clones in the CF population.

## Supporting Information

S1 Fig Empirical cumulative distribution functions (ECDF) of various phenotypic of *P*. *aeruginosa* isolates. For each group (Prairie Epidemic Strain, PES, red; other epidemic strains, OES, blue;, and local non-epidemic isolates, green), the line plots the cumulative percentage of isolates from that group with at least as much activity as its location on the x-axis. Each step of the line represents an additional value, which generally corresponds to a single isolate but in cases where multiple isolates exhibited the same amount of activity (ie. null activity) the line increases by larger vertical steps. The horizontal distance of each step is the difference between each subsequent value. The Kolomogorov-Smirnov (KS) test was used to determine if the ECDFs of each group were significantly different. This test determined whether values from one group tended to be lower than those of another group. By checking each pairwise comparison for each phenotypic trait, significant differences between each group could be determined. X-axes for swarming, swimming, biofilm biomass, biofilm growth, and planktonic growth were log transformed to improve readability of the graph but this transformation had no effect on the data or the KS test.
(PDF)

S2 Fig Percent of isolates with the mucoid phenotype in each group. A) PES. B) OES. C) Local Isolates. Activity was scored as positive or negative on *Pseudomonas* Isolation Agar plates and percentages were calculated within each group type.
(PDF)

S3 Fig Swarm and swim motility assays of *P*. *aeruginosa* isolates. Isolates were separated into three groups for comparative analysis: Prairie Epidemic Strain (PES, red), other epidemic strains (OES, blue), and local non-epidemic isolates (green) groups. Each circle depicts the mean activity for one *P*. *aeruginosa* isolate. The horizontal line indicates the mean activity of each group whereas the error bars indicate the standard deviation.
(PDF)

S4 Fig *P*. *aeruginosa* hemolysis activity on 5% sheep blood for each group. Hemolytic activity was scored as β- (complete lysis), α- (partial lysis), or γ- (no lysis) hemolysis.
(PDF)

S1 Table Amplification and sequencing primers used for *P*. *aeruginosa* MLST. (PDF)

S2 Table MLST analysis of *P*. *aeruginosa* isolates. PES: Prairie Epidemic Strain. The other epidemic strains are composed of the Liverpool Epidemic Strain (LES)/Strain A, Strain B, Midlands 1 (Md1), Manchester Epidemic Strain (MES), and distinct Australian Epidemic Strains (AUST). Local *P*. *aeruginosa* isolates were collected from the Calgary Adult CF Clinic. The PES and local isolates were also separated into early or late groups based on when they were isolated. “New” denotes a novel allele type/sequence type.
(PDF)

S3 Table Results from Kolmogorov-Smirnov (KS) test comparing each pairwise combination of *P*. *aeruginosa* groups. KS uses the empirical cumulative distribution function (ECDF) and determines whether values from one group tend to be larger or smaller than values from the other group. KS tests were performed to check whether the ECDF of the first group listed in the table was larger or smaller than that of the second group. P-values were considered significant (red text) if they were below a Benjamini-Hochberg corrected false discovery rate (FDR) cut-off of 0.05. Corrected cut-offs were KS larger ≤ 0.0106 and KS smaller ≤ 6.79x10^-3^. In the case of non-significance for the KS tests, both p-values are reported.
(PDF)

S4 Table Chi-square test comparing *P*. *aeruginosa* isolates from each group. Mucoidy was assessed on the ability of isolates to form mucoid colonies on *Pseudomonas* isolation agar. β- (complete lysis) and α- (partial lysis) was group together as one category and compared against the γ- (no lysis) type. P-value cutoff was set at 0.05 with degree of freedom of 1. Significant differences are indicated in red text.
(PDF)

S5 Table Mann-Whitney U (MWU) tests comparing various virulence factors of longitudinally-collected isolates from the same patient. P-values reported here were considered significant (in red) if they were below a Benjamini-Hochberg FDR adjusted cutoff of 0.05 (p ≤ 4.15x10^-3^).
(PDF)

## Abbreviations

AUST: Australian Epidemic Strain
LES: Liverpool Epidemic Strain
MES: Manchester Epidemic Strain
Md1: Midlands 1 strain
OES: Other Epidemic Strains
PES: Prairie Epidemic Strain
