# Supplementary figures and images for: Phenotypic and Genotypic Comparison of Epidemic and Non-Epidemic Strains of Pseudomonas aeruginosa from Individuals with Cystic Fibrosis
Source: PLoS One. 2015 Nov 23;10(11):e0143466. doi: 10.1371/journal.pone.0143466 (PMC4657914; doi:10.1371/journal.pone.0143466)

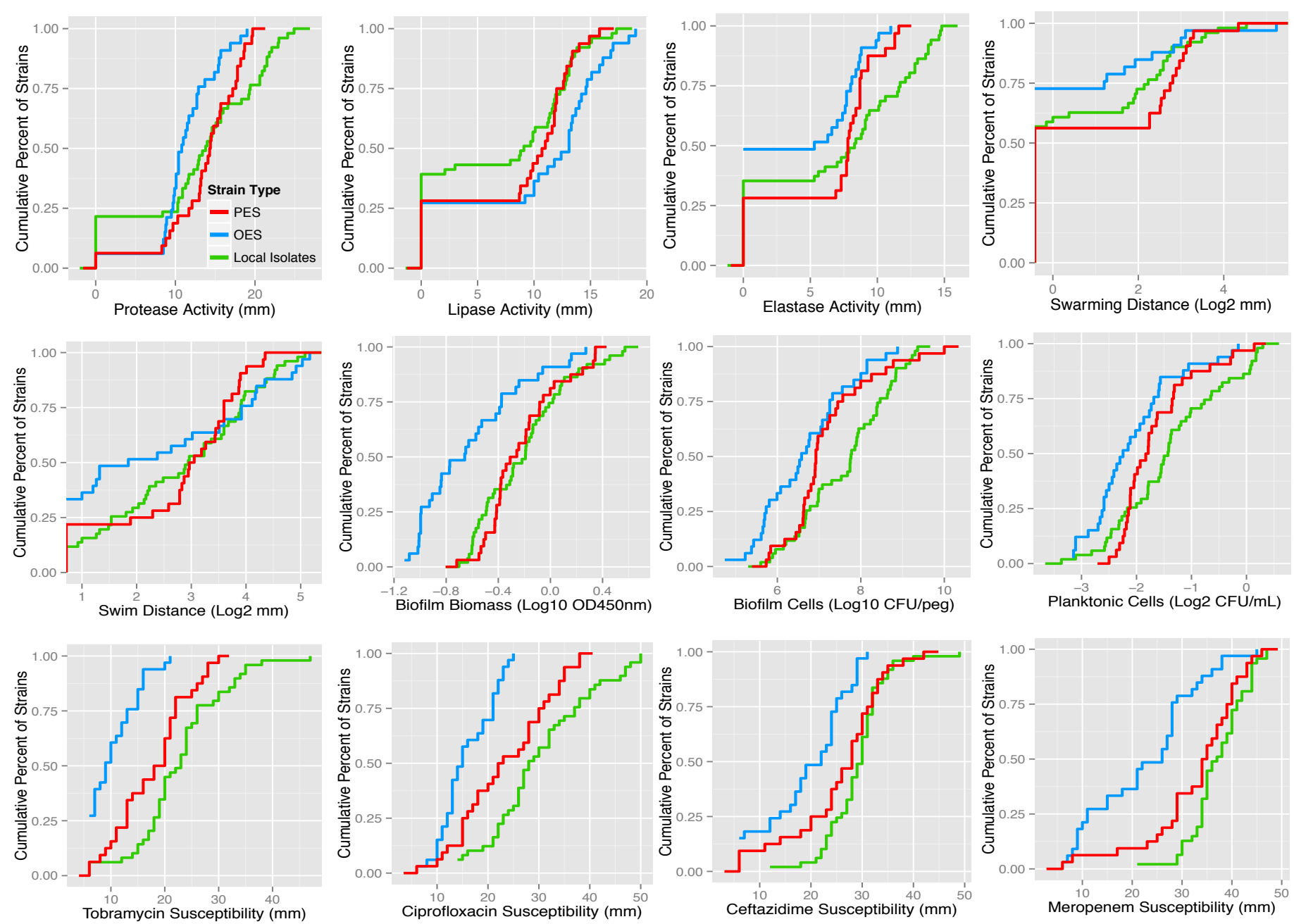

Supplement: S1 Fig — For each group (Prairie Epidemic Strain, PES, red; other epidemic strains, OES, blue;, and local non-epidemic isolates, green), the line plots the cumulative percentage of isolates from that group with at least as much activity as its location on the x-axis. Each step of the line represents an additional value, which generally corresponds to a single isolate but in cases where multiple isolates exhibited the same amount of activity (ie. null activity) the line increases by larger vertical steps. The horizontal distance of each step is the difference between each subsequent value. The Kolomogorov-Smirnov (KS) test was used to determine if the ECDFs of each group were significantly different. This test determined whether values from one group tended to be lower than those of another group. By checking each pairwise comparison for each phenotypic trait, significant differences between each group could be determined. X-axes for swarming, swimming, biofilm biomass, biofilm growth, and planktonic growth were log transformed to improve readability of the graph but this transformation had no effect on the data or the KS test. (PDF) [file pone.0143466.s001.pdf]

**A) PES**

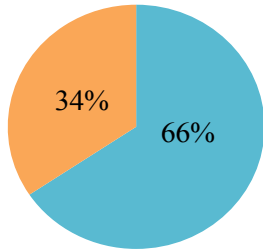

**B) OES**

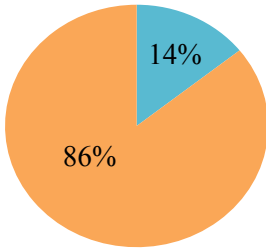

**C) Local Isolates**

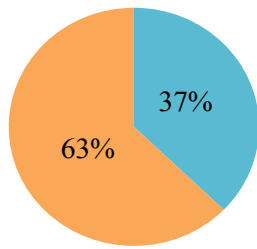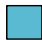

**Mucooid**

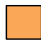

**Non-Mucooid**

Supplement: S2 Fig — A) PES. B) OES. C) Local Isolates. Activity was scored as positive or negative on Pseudomonas Isolation Agar plates and percentages were calculated within each group type. (PDF) [file pone.0143466.s002.pdf]

A

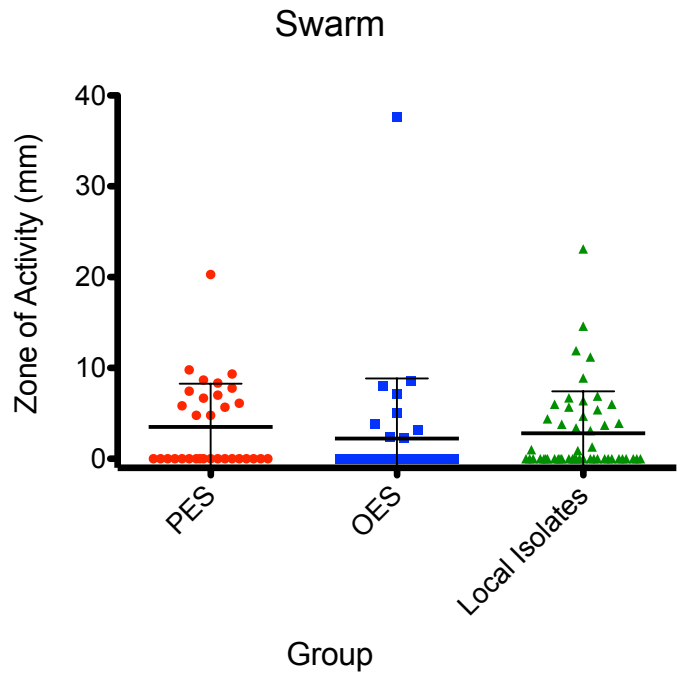

B

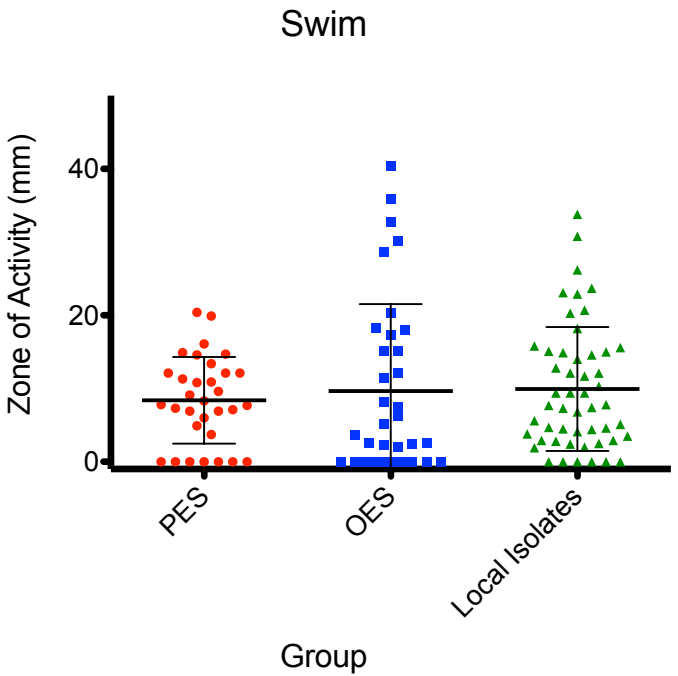

Supplement: S3 Fig — Isolates were separated into three groups for comparative analysis: Prairie Epidemic Strain (PES, red), other epidemic strains (OES, blue), and local non-epidemic isolates (green) groups. Each circle depicts the mean activity for one P. aeruginosa isolate. The horizontal line indicates the mean activity of each group whereas the error bars indicate the standard deviation. (PDF) [file pone.0143466.s003.pdf]

**PES**

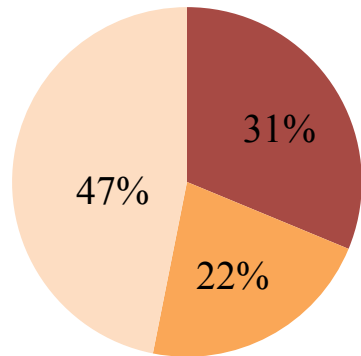

**OES**

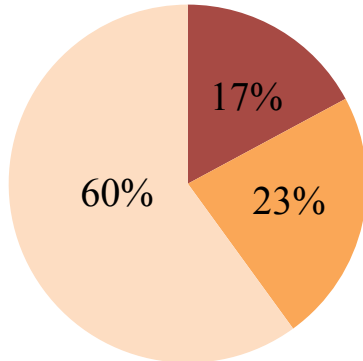

**Local Isolates**

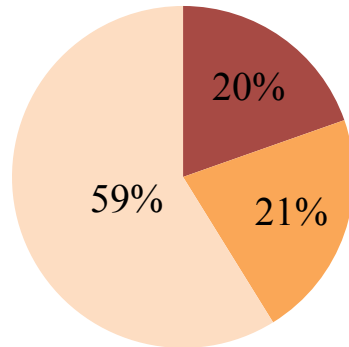

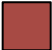  $\beta$

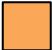  $\alpha$

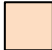  $\gamma$

Supplement: S4 Fig — Hemolytic activity was scored as β- (complete lysis), α- (partial lysis), or γ- (no lysis) hemolysis. (PDF) [file pone.0143466.s004.pdf]
