# Supplementary material for: Phenotypic and Genotypic Comparison of Epidemic and Non-Epidemic Strains of Pseudomonas aeruginosa from Individuals with Cystic Fibrosis
Source: PLoS One. 2015 Nov 23;10(11):e0143466. doi: 10.1371/journal.pone.0143466 (PMC4657914; doi:10.1371/journal.pone.0143466)
Supplement: S1 Table — (PDF) [file pone.0143466.s005.pdf]

Locus	Function	Forward (5' to 3')	Reverse (5' to 3')	Amplicon size (bp)
acsA	Amplification	ACCTGGTGTACGCCTCGCTGAC	GACATAGATGCCCTGCCCCTTGAT	842
	Sequencing	GCCACACCTACATCGTCTAT	GTTGCCGAGGTTGCCAC	430
aroE	Amplification	TGGGGCTATGACTGGAAACC	TAACCCGGTTTTGTGATTCCTACA	1053
	Sequencing	ATGTCACCGTGCCGTTCAAG	TGAAGGCAGTCGGTTCCTTG	498
guaA	Amplification	CGGCCTCGACGTGTGGATGA	GAACGCCTGGCTGGTCTTGTGGTA	940
	Sequencing	AGGTCGGTTCCTCCAAGGTC	GACGTTGTGGTGCGACTTGA	413
mutL	Amplification	TGGCCAAGGAACTCCTGGAA	GGCCGGGTATAGGCGGAATA	1123
	Sequencing	AGAAGACCGAGTTCGACCAT	GGTGCCATAGAGGAAGTCAT	482
nuoD	Amplification	ACCGCCACCCGTATCTG	TCTCGCCCATCTTGACCA	1042
	Sequencing	ACGGCGAGAACGAGGACTAC	GCGGTCGGTGAAGGTGAA	404
ppsA	Amplification	GGTCGCTCGGTCAAGGTAGTGG	GGGTTCTCTTCTTCCGGCTCGTAG	989
	Sequencing	GGTGACGACGGCAAGCTGTA	TATCGCCTTCGGCACAGG	410
trpE	Amplification	GCGGCCCAGGGTCGTGAG	CCCGGCGCTTGTTGATGGTT	811
	Sequencing	TTCAACTTCGGCGACTTCCA	GGTGTCCATGTTGCCGTTCC	483
