# Supplementary material for: Phenotypic and Genotypic Comparison of Epidemic and Non-Epidemic Strains of Pseudomonas aeruginosa from Individuals with Cystic Fibrosis
Source: PLoS One. 2015 Nov 23;10(11):e0143466. doi: 10.1371/journal.pone.0143466 (PMC4657914; doi:10.1371/journal.pone.0143466)
Supplement: S2 Table — PES: Prairie Epidemic Strain. The other epidemic strains are composed of the Liverpool Epidemic Strain (LES)/Strain A, Strain B, Midlands 1 (Md1), Manchester Epidemic Strain (MES), and distinct Australian Epidemic Strains (AUST). Local P. aeruginosa isolates were collected from the Calgary Adult CF Clinic. The PES and local isolates were also separated into early or late groups based on when they were isolated. “New” denotes a novel allele type/sequence type. (PDF) [file pone.0143466.s006.pdf]

				Allele Type							
**Patient**	**Strain**	**Early/ Late**	**Type**	**acsA**	**aroE**	**guaA**	**mutL**	**nuoD**	**ppsA**	**trpE**	**Sequence Type**
A12	233	Early	PES Stable	1	5	7	5	4	4	2	192
	234	Early	PES Stable	1	5	132	5	4	4	2	1495
	24	Late	PES Stable	1	5	7	5	4	4	2	192
	25	Late	PES Stable	1	5	7	5	4	4	2	192
A25	590	Early	PES Stable	1	5	7	5	4	4	2	192
	591	Early	PES Stable	1	5	7	5	4	4	2	192
	592	Early	PES Stable	1	5	7	5	4	4	2	192
	47	Late	PES Stable	1	5	7	5	4	4	2	192
	48	Late	PES Stable	1	5	7	5	4	4	2	192
A32	222	Early	PES Stable	1	5	7	5	4	4	2	192
	60	Late	PES Stable	1	5	7	5	4	4	2	192
	61	Late	PES Stable	1	5	7	5	4	4	2	192
	62	Late	PES Stable	1	5	7	5	4	4	2	192
A41	577	Early	PES Stable	1	5	7	5	4	4	2	192
	578	Early	PES Stable	1	5	7	5	4	4	2	192
	79	Late	PES Stable	1	5	7	5	4	4	2	192
	80	Late	PES Stable	1	5	7	5	4	4	2	192
A97	622	Early	PES Stable	1	5	7	5	4	4	2	192
	623	Early	PES Stable	1	5	7	5	4	4	2	192
	173	Late	PES Stable	1	5	7	5	4	4	2	192
	174	Late	PES Stable	1	5	7	5	4	4	2	192
	175	Late	PES Stable	1	5	7	5	4	4	2	192
	176	Late	PES Stable	1	5	7	5	4	4	2	192
---	22A	---	Strain A	6	5	11	3	4	23	1	146
---	27A	---	Strain A	6	5	11	3	4	23	1	146
---	56A	---	Strain A	6	5	11	3	4	4	1	683
---	19A	---	Strain A	6	5	11	3	4	4	1	683
---	54A	---	Strain A	6	5	11	3	4	23	1	146
---	63A	---	Strain A	6	5	11	3	4	23	1	146

---	132A	---	Strain A	6	5	11	3	4	23	1	146
---	9A	---	Strain A	6	5	11	3	4	23	1	146
---	29A	---	Strain A	6	5	11	3	4	23	1	146
---	53A	---	Strain A	6	5	11	3	4	23	1	146
---	201	---	LES	6	5	11	3	4	23	1	146
---	203	---	LES	6	5	11	3	4	23	1	146
---	219	---	LES	Untypeable by MLST							
---	220	---	LES	6	5	11	3	4	23	1	146
---	228	---	LES	6	5	11	3	4	23	1	146
---	229	---	LES	6	5	11	3	4	23	1	146
---	119B	---	Strain B	6	68	20	11	4	4	7	439
---	5B	---	Strain B	6	68	20	11	4	4	7	439
---	3-9B	---	Strain B	11	5	New	3	4	4	61	New
---	7B	---	Strain B	6	68	20	11	4	4	7	439
---	20B	---	Strain B	Untypeable by MLST							
---	6-9B	---	Strain B	6	68	20	11	4	4	7	439
---	23B	---	Strain B	6	68	20	11	4	4	7	439
---	8B	---	Strain B	6	68	20	11	4	4	7	439
---	204	---	Md1	17	5	1	3	13	6	7	148
---	208	---	Md1	17	5	1	3	13	6	7	148
---	222	---	MES	28	5	11	18	4	13	7	New
---	AUST-01RA	---	AUST-01	11	84	11	3	4	4	7	649
---	AUST-01RB	---	AUST-01	11	84	11	3	4	4	7	649
---	209	---	AUST-01	11	84	11	3	4	4	7	649
---	AUST-02A	---	AUST-02	28	5	11	5	4	4	7	775
----	AUST-02B	---	AUST-02	28	5	11	5	4	4	7	775
---	AUST-03	---	AUST-03	28	5	5	11	3	15	44	242
---	AUST-04	---	AUST-04	16	125	30	72	4	13	7	787
---	P42	---	AUST-06	17	126	11	3	11	12	111	New
A2	588	Early	Local Stable	11	5	11	11	3	6	27	569
	589	Early	Local Stable	11	5	11	11	3	6	27	569
	3	Late	Local Stable	11	5	11	11	3	6	27	569
A9	240	Early	Local Stable	28	5	11	5	15	12	7	807
	19	Late	Local Stable	28	5	11	5	15	12	7	807
	20	Late	Local Stable	28	5	11	5	15	12	7	807
A14	210	Early	Local Stable	11	150	11	7	27	6	7	1207
	211	Early	Local Stable	11	150	11	7	27	6	7	1207
	27	Late	Local Stable	11	150	11	7	27	6	7	1207
	28	Late	Local Stable	11	150	11	7	27	6	7	1207
A18	602	Early	Local	36	27	28	3	4	13	7	179

			Stable								
	603	Early	Local Stable	36	27	28	3	4	13	7	179
	34	Late	Local Stable	36	176	28	150	4	13	176	1745
	35	Late	Local Stable	147	27	28	3	4	13	7	1494
	36	Late	Local Stable	36	27	28	3	4	13	7	179
A34	595	Early	Local Stable	36	5	36	3	3	13	7	1499
	66	Late	Local Stable	28	5	36	3	3	13	7	155
	67	Late	Local Stable	28	5	36	3	3	13	7	155
A35	247	Early	Local Stable	11	22	New	11	4	15	19	New
	68	Late	Local Stable	11	22	1	11	4	15	19	New
A40	625	Early	Local Stable	16	22	5	11	4	6	10	609
	77	Late	Local Stable	16	22	5	11	4	6	10	609
	78	Late	Local Stable	16	22	5	11	4	6	10	609
A51	583	Early	Local Stable	6	5	1	4	4	6	17	1747
	95	Late	Local Stable	6	5	1	4	4	6	17	1747
A52	584	Early	Local Stable	36	27	28	3	4	13	7	179
	585	Early	Local Stable	36	27	28	3	4	13	7	179
	97	Late	Local Stable	36	27	28	3	4	13	7	179
A64	243	Early	Local Stable	13	4	5	5	12	7	15	308
	244	Early	Local Stable	13	4	5	5	12	7	15	308
	119	Late	Local Stable	13	4	5	5	12	7	172	1746
	120	Late	Local Stable	Untypeable by MLST							
A85	630	Early	Local Stable	16	5	30	New	4	13	7	New
	153	Late	Local Stable	16	5	30	11	4	13	7	1748
A129	637	Early	Local Stable	36	27	28	3	4	13	7	179
	638	Early	Local Stable	36	27	28	3	4	13	7	179
	639	Early	Local Stable	36	27	28	3	4	13	7	179
	284	Late	Local Stable	36	27	28	148	4	13	7	1496
A11	604	Early	Displaced	23	5	11	7	1	12	7	274

			Local								
	23	Late	Replace-ment PES	1	5	7	5	4	4	2	192
A43	261	Early	Displaced Local	6	5	6	7	4	6	7	27
	83	Late	Replace-ment PES	1	5	7	5	4	4	2	192
	84	Late	Replace-ment PES	1	5	7	5	4	4	2	192
A78	568	Early	Displaced Local	11	20	1	65	4	4	10	381
	569	Early	Displaced Local	11	20	1	65	4	4	10	381
	570	Early	Displaced Local	11	20	1	65	4	4	10	381
	571	Early	Displaced Local	17	22	5	3	1	14	3	389
	142	Late	Replace-ment PES	1	5	7	5	4	4	2	192
	143	Late	Replace-ment PES	1	5	7	5	4	4	2	192
A131	290	Early	Displaced Local	39	5	4	7	3	15	2	1497
	291	Late	Replace-ment PES	1	5	7	5	4	4	2	192
A134	381	Early	Displaced Local	7	5	12	3	4	1	7	549
	382	Early	Displaced Local	7	5	12	3	4	1	7	549
	383	Late	Replace-ment PES	1	5	7	5	4	4	2	192
	384	Late	Replace-ment PES	1	5	7	5	4	4	2	192
	385	Late	Replace-ment PES	1	5	7	5	4	4	2	192
A8	654	Early	Displaced Local	16	5	20	5	4	4	10	1498
	16	Late	Replace-ment Local	23	5	11	7	1	12	7	274
	17	Late	Replace-ment Local	23	5	11	7	1	12	7	274
	18	Late	Replace-ment Local	23	5	11	7	1	12	7	274
