# Supplementary material for: Phenotypic and Genotypic Comparison of Epidemic and Non-Epidemic Strains of Pseudomonas aeruginosa from Individuals with Cystic Fibrosis
Source: PLoS One. 2015 Nov 23;10(11):e0143466. doi: 10.1371/journal.pone.0143466 (PMC4657914; doi:10.1371/journal.pone.0143466)
Supplement: S3 Table — KS uses the empirical cumulative distribution function (ECDF) and determines whether values from one group tend to be larger or smaller than values from the other group. KS tests were performed to check whether the ECDF of the first group listed in the table was larger or smaller than that of the second group. P-values were considered significant (red text) if they were below a Benjamini-Hochberg corrected false discovery rate (FDR) cut-off of 0.05. Corrected cut-offs were KS larger ≤ 0.0106 and KS smaller ≤ 6.79x10^-3^. In the case of non-significance for the KS tests, both p-values are reported. (PDF) [file pone.0143466.s007.pdf]

Assay	PES vs OES		PES vs Local Isolates		OES vs Local Isolates	
	**p-value**	**Group with higher activity**	**p-value**	**Group with higher activity**	**p-value**	**Group with higher activity**
**Protease**	**$3.25x10^{-4}$**	PES	0.113<br>0.322	---	**0.0106**	Local Isolates
**Elastase**	1<br>0.0335	---	0.0652<br>0.512	---	**$3.93x10^{-3}$**	Local Isolates
**Lipase**	**$6.76x10^{-3}$**	OES	0.914<br>0.322	---	**$6.79x10^{-3}$**	OES
**Swarm**	0.973<br>0.0549	---	0.916<br>0.200	---	0.255<br>0.967	---
**Swim**	0.197<br>0.0539	---	0.360<br>0.412	---	0.0307<br>0.640	---
**Biofilm Biomass**	**$2.01x10^{-5}$**	PES	0.590<br>0.218	---	**$2.67x10^{-5}$**	Local Isolates
**Biofilm Growth**	1<br>0.0281	---	**$4.66x10^{-3}$**	Local Isolates	**$1.92x10^{-3}$**	Local Isolates
**Planktonic Growth**	0.923<br>0.0106	---	0.0202<br>0.496	---	**$1.92x10^{-3}$**	Local Isolates
**Tobramycin Susceptibility**	**$5.00x10^{-4}$**	PES	0.0177<br>1	---	**$9.95x10^{-11}$**	Local Isolates
**Ceftazidime Susceptibility**	1<br>0.0116	---	0.184<br>0.978	---	**$8.51x10^{-6}$**	Local Isolates
**Ciprofloxacin Susceptibility**	**$3.47x10^{-3}$**	PES	0.0442<br>1	---	**$6.46x10^{-8}$**	Local Isolates
**Meropenem Susceptibility**	**$3.67x10^{-5}$**	PES	0.0506<br>1	---	**$1.57x10^{-10}$**	Local Isolates
