# Supplementary material for: Phenotypic and Genotypic Comparison of Epidemic and Non-Epidemic Strains of Pseudomonas aeruginosa from Individuals with Cystic Fibrosis
Source: PLoS One. 2015 Nov 23;10(11):e0143466. doi: 10.1371/journal.pone.0143466 (PMC4657914; doi:10.1371/journal.pone.0143466)
Supplement: S4 Table — Mucoidy was assessed on the ability of isolates to form mucoid colonies on Pseudomonas isolation agar. β- (complete lysis) and α- (partial lysis) was group together as one category and compared against the γ- (no lysis) type. P-value cutoff was set at 0.05 with degree of freedom of 1. Significant differences are indicated in red text. (PDF) [file pone.0143466.s008.pdf]

	PES vs OES	OES vs Local Isolates	PES vs Local Isolates
**Mucoidy**	**18.554**	**5.443**	**6.339**
**Hemolysis**	1.158	1.131	0.0119
