# Supplementary material for: Phenotypic and Genotypic Comparison of Epidemic and Non-Epidemic Strains of Pseudomonas aeruginosa from Individuals with Cystic Fibrosis
Source: PLoS One. 2015 Nov 23;10(11):e0143466. doi: 10.1371/journal.pone.0143466 (PMC4657914; doi:10.1371/journal.pone.0143466)
Supplement: S5 Table — P-values reported here were considered significant (in red) if they were below a Benjamini-Hochberg FDR adjusted cutoff of 0.05 (p ≤ 4.15x10^-3^). (PDF) [file pone.0143466.s009.pdf]

Assay	Strain Replacement	Stable PES	Stable Local Isolate
**Protease**	**$7.87x10^{-4}$**	0.251	**$1.29x10^{-4}$**
**Elastase**	**$1.6x10^{-3}$**	0.303	0.0278
**Lipase**	0.0768	0.593	0.666
**Swarm**	0.559	0.231	0.222
**Swim**	0.0321	0.641	0.0364
**Biofilm Biomass**	0.0503	0.208	0.0773
**Biofilm Growth**	0.0422	0.563	0.122
**Planktonic Growth**	0.133	0.321	0.163
**Tobramycin**	**$4.15x10^{-3}$**	0.618	0.0792
**Ceftazidime**	0.266	0.367	0.781
**Ciprofloxacin**	**$6.99x10^{-4}$**	0.619	**$9.54x10^{-4}$**
**Meropenem**	0.0335	**$7.92x10^{-4}$**	0.546
